# Supplementary figures and images for: A Platform for Cognitive Monitoring of Neurosurgical Patients During Hospitalization
Source: Front Hum Neurosci. 2021 Nov 22;15:726998. doi: 10.3389/fnhum.2021.726998 (PMC8645698; doi:10.3389/fnhum.2021.726998)

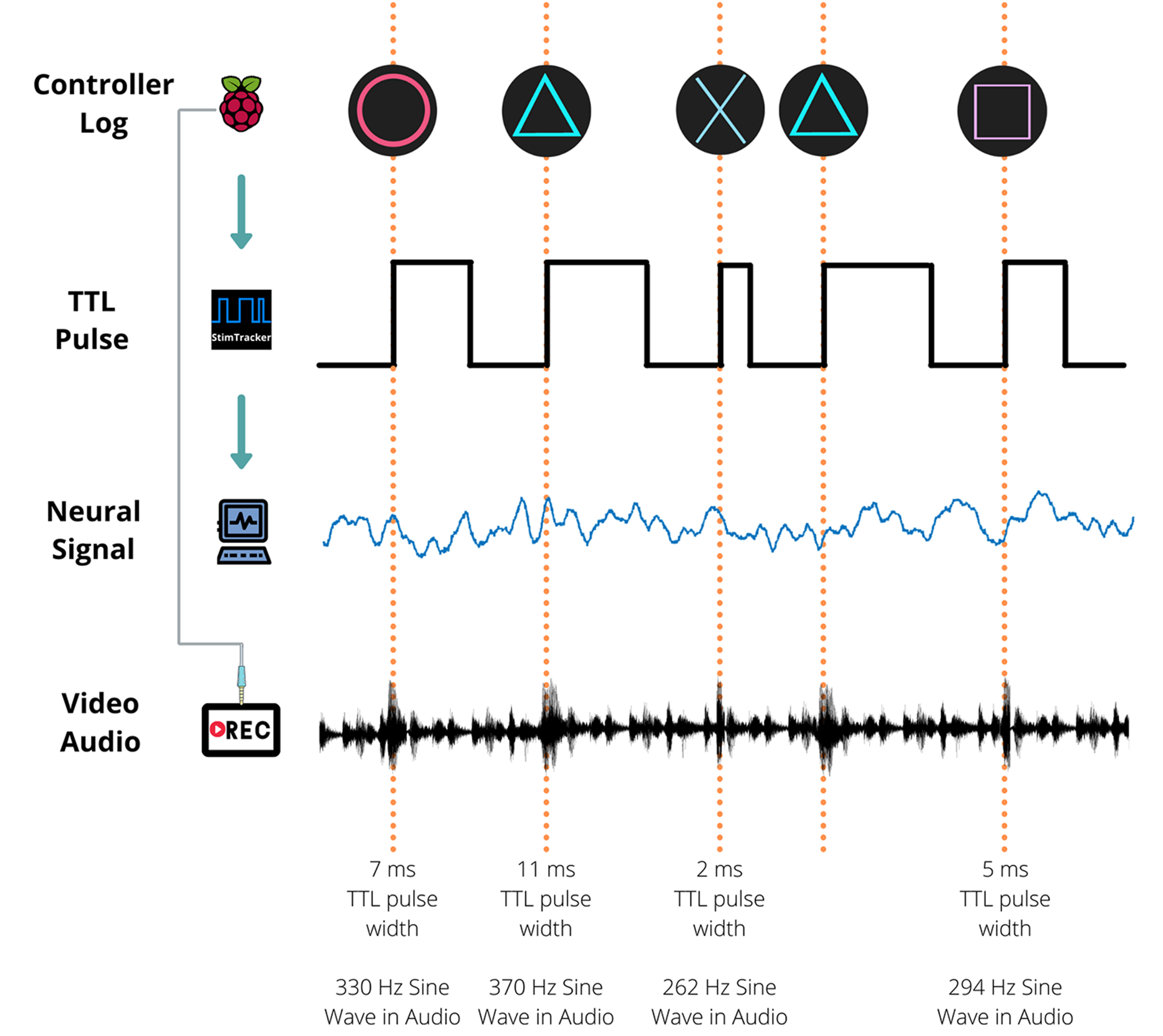

Supplement: Supplementary Figure 1 — Synchronization process. Controller button presses are logged on the Raspberry Pi. Each button sends a unique sine wave tone in the video audio and a unique TTL pulse that is aligned with the neural signal. The controller log, TTL pulses, and the embedded tones in the video audio allow for offline synchronization between the PS4 system and the neural recordings. [file Image_1.TIFF]

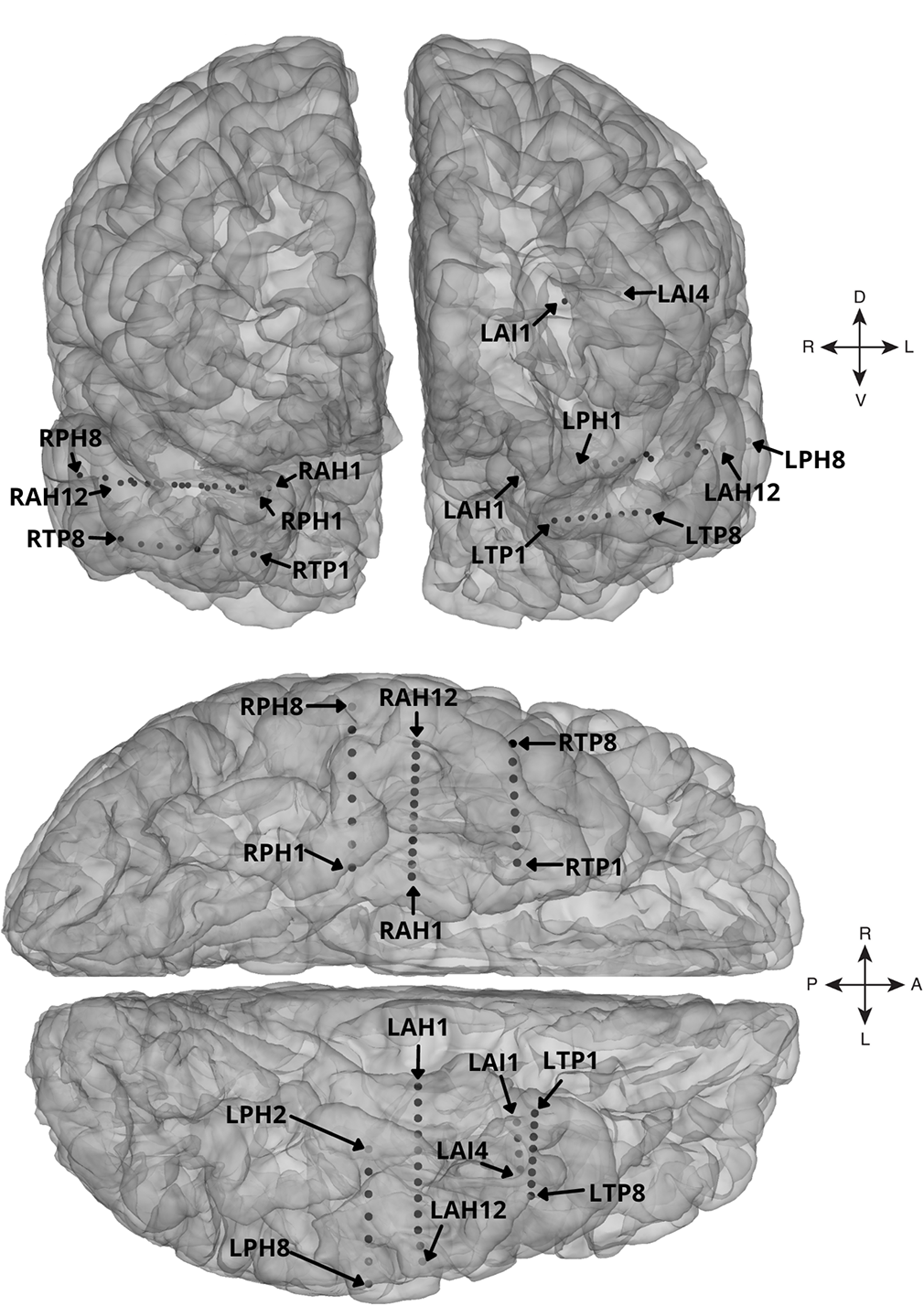

Supplement: Supplementary Figure 2 — Electrode localization from the patient analyzed with the acoustic encoding model. [file Image_2.TIFF]
